# Multiple primary pulmonary meningioma: A case report and literature review

**DOI:** 10.1111/1759-7714.14542

**Published:** 2022-06-22

**Authors:** Xin Huang, Yun‐Fei Mou, Fu‐Qiang Ren, Yuan Wang, Yi Yang

**Affiliations:** ^1^ Department of Thoracic Surgery, Chengdu Third People's Hospital the Affiliated Hospital of Southwest Jiaotong University Chendu China

## Abstract

Lung cancer has become a leading cause of cancer‐related deaths. With the conventional use of low‐dose spiral computed tomography (CT) in physical examinations, an increasing number of small pulmonary nodules are screened. However, primary pulmonary meningiomas (PPMs) are rarely reported. Here, we report the case of a 64‐year‐old woman who had a CT scan during physical examination, which revealed three ground‐glass‐like opacity pulmonary nodules in both lungs. The patient underwent video‐assisted thoracoscopic wedge resection of the right upper and lower lobes. Paraffin sections revealed pulmonary meningothelial‐like and collagenous nodules in the right upper and lower lobes which stained as follows: EMA+, VIM+, SMA‐, S‐100‐, CD34‐, STAT6‐, Ki‐67+ (2%), and CgA‐. Primary pulmonary meningiomas (PPMs) were finally diagnosed. PPM is a kind of rare and benign tumor. Surgery can provide a precise pathological examination, and patients can achieve an excellent prognosis after surgical resection.

## Graphical abstract

Multiple ground‐glass nodules were dectected in both lungs by chest computed tomography. The two nodules in the right lung were wedge resected by thoracoscopic surgery, and were confirmed as primary pulmonary meningioma by HE staining and immunohistochemistry.

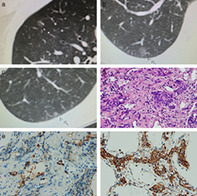

## INTRODUCTION

Lung cancer has become a leading cause of cancer‐related deaths. With the conventional use of low‐dose spiral computed tomography (CT) scanning in physical examinations, an increasing number of small pulmonary nodules are screened. However, primary pulmonary meningiomas (PPMs) are rarely reported.

## CASE REPORT

**FIGURE 1:**
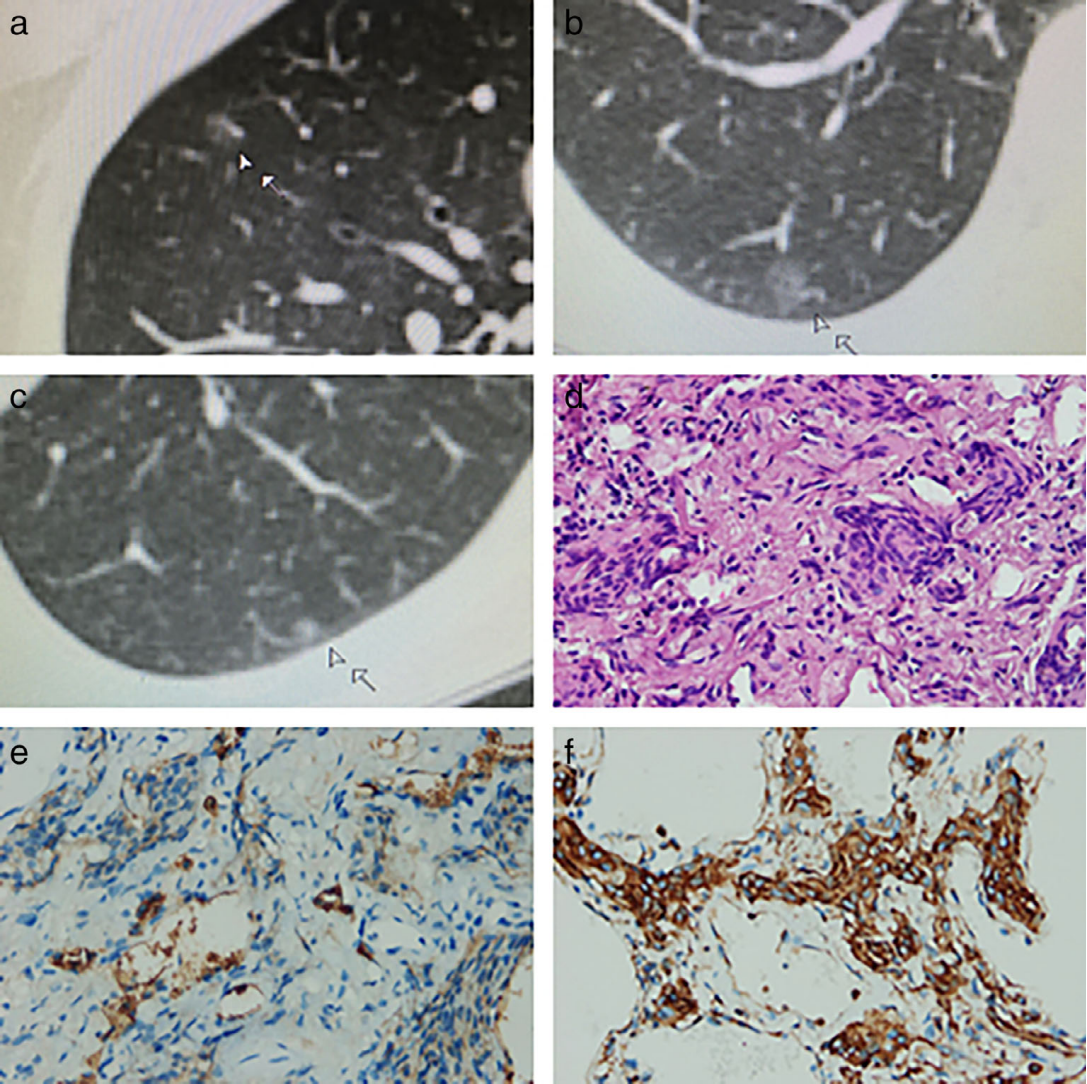
(a–c) The nodules located in the right upper/right lower/left lower lobes. (d) Hematoxylin and eosin (HE) staining showed many pulmonary meningothelial‐like nodules (400× magnification). (e) Immunohistochemical staining with epithelial membrane antigen (EMA) was positive (400× magnification). (f) Immunohistochemical staining with vimentin (VIM) was positive (400× magnification)

A 64‐year‐old woman was referred to our department because of pulmonary nodules in both lungs which had previously been identified during a physical examination 2 years previously. The patient had an 8 month history of hypertension, but no history of smoking. The patient's younger brother and daughter had suffered from lung cancer and adenocarcinoma in situ, respectively. A computed tomography (CT) scan revealed three ground‐glass nodules in both lungs (right upper/right lower/left lower lobes), which were similar to multicentric early‐stage adenocarcinoma (Figure 1a–c). Both head and postoperative enhanced magnetic resonance imaging (MRI) showed only a few cerebral ischemic foci in the left centrum semiovale and mild demyelination of white matter. No intracranial meningioma was observed, and tumor marker levels were all normal. The patient exhibited no symptoms and the nodules showed no change after oral antibiotics for 2 weeks. In addition, she was anxious because of her positive family history and CT findings. After multidisciplinary discussion, video‐assisted thoracoscopic surgery (VATS) was performed. The patient underwent successful VATS wedge resection of the right upper and lower lobe nodules.

Intraoperative pathological examination of the snap‐frozen sections revealed benign pulmonary lesions. Postoperative paraffin sections revealed pulmonary meningothelial‐like and collagenous nodules in the right upper and lower lobe, which stained as follows: EMA+, VIM+, SMA‐, S‐100‐, CD34‐, STAT6‐, Ki‐67+ (2%), and CgA‐ (Figure 1d–f). Finally, PPM was diagnosed.

## DISCUSSION

Meningiomas are relatively common tumors of the central nervous system. The incidence of an ectopic meningioma is 1%–2% of meningiomas, and the lungs are an uncommon site.^1^ Primary pulmonary meningiomas (PPM) often occur in middle‐aged or older individuals, with a slight predominance in women. Weiss et al. reported the first case of PPM, identifying it as a rare, slow‐growing tumor.^2^ Since then, few individual cases have been reported globally. Most PPMs are benign lesions that grow slowly, and patients are usually asymptomatic. They are often found during physical examinations. Malignant PPMs with aggressive growth and distant metastases are extremely rare. At present, only five cases have been confirmed pathologically in the literature, including three confirmed by pathological examinations after lung resection, one by autopsy, and one by percutaneous lung biopsy.^3^, ^4^, ^5^, ^6^

PPM mostly manifests as a single solitary pulmonary nodule.^7^, ^8^, ^9^ Previous case reports have not reported on the specific density or appearance of the nodules. In our case, multiple nodules in both lungs of the patient had a ground‐glass‐like opacity; the right upper lobe nodule had a density of −657 (diameter: 5.5 mm), and the right lower lobe nodule had a density of −748 (diameter: 8.8 mm). The left upper lobe nodule had a density of −568 and measured 4.1 mm. As these measurements are in line with early‐stage malignant pulmonary tumors, atypical adenomatous hyperplasia or adenocarcinoma in situ was preoperatively suspected.

Because of the morphological PPM diversity on CT images, clinical diagnosis is extremely challenging. Early lesions can manifest as ground glass‐like changes when they are small, which can easily be confused with early‐stage lung cancers. As the lesions grow, it becomes difficult to distinguish them from other benign lung tumors, such as pulmonary hamartoma, special infections (such as cryptococcal infection,virus infection) and lipomas. It is also difficult to distinguish PPMs with aggressive growth or multiple metastases from advanced lung cancers. Meanwhile, CT imaging cannot solely guide a patient's treatment as it provides no specificity. Moreover, previously reported cases have indicated that positron emission tomography‐computed tomography (PET‐CT) use during examination also provides no advantage in the diagnosis of PPM, so that the necessity of a pathological examination after surgery in our case was the best choice.^10^, ^11^

The majority of PPM lesions are benign and have an excellent prognosis after surgical resection, and Spinelli et al. reported a PPM case with an 8‐year nonrecurrence after a pulmonary wedge resection.^12^ However, because a precise diagnosis is nearly impossible before surgery, the choice of treatment mainly depends on the number of lesions and their location, and partial or complete resection of the lesions may serve as an effective treatment and precise diagnosis.

In conclusion, PPM is a kind of rare and benign tumor, but only five cases of malignant PPMs have previously been reported. The diagnosis of PPM relies on pathological examination, and surgery can provide a precise diagnosis and excellent prognosis.

## CONFLICT OF INTEREST

The authors confirm that there are no conflicts of interest.

## References

[1] Xu KK , Tian F , Cui Y . Primary pulmonary meningioma presenting as a micro solid nodule: a rare case report. Thor Cancer. 2018;9(7):874–6. 10.1111/1759-7714.12639 PMC6026600 29718593

[2] Weis A , Philippides D , Montrieul B , Steimle R . Primary pulmonary meningioma: a rare, slow‐growing tumor. Radiol Electrol Arch Electr Medicale. 1952;4:701–3. 13045242

[3] Kenmitz P , Spormann H , Heinrich P . Meningioma of lung:first report with light and electron microscopic findings. Ultrastruct Pathol. 1982;3(4):359–65. 7157498 10.3109/01913128209018558

[4] Prayson RA , Farver CF . Primary pulmonary malignant meningioma. Am J Surg Pathol, 1999, 23(6): 722–726. 10366156 10.1097/00000478-199906000-00013

[5] Van der Meij JJ , Boomars KA , van den Bancosch JM , Van Boven WJ , De Bruin PC , Seldenrijk CA . Primary pulmonary malignant meningioma. Ann Thorac Surg, 2005, 80(4): 1523–1525. 16181912 10.1016/j.athoracsur.2004.04.015

[6] Weber C , Pautex S , Zulian GB , Pusztaszeri M , Lobrinus JA Primary pulmonary malignant meningioma with lymph node and liver metastasis in a centenary woman, an autopsy case. Virchows Arch. 2013, 462(4): 481–485. 23443940 10.1007/s00428-013-1383-7

[7] Gürçay N , Öztürk A , Demirağ F , İncekara F . Primary pulmonary meningioma mimicking pulmonary metastasis: a rare case report. Turk Gogus Kalp Damar Cerrahisi Derg. 2020;28(4):699–701. 33403148 10.5606/tgkdc.dergisi.2020.19370 PMC7759054

[8] Baş A , Valiyev E , Özkan N , Tombul L , Yonat S , Sayan M . et al. A rare entity: primary pulmonary meningioma. Turk J Pathol. 2021;10: 5. Online ahead of print. 10.5146/tjpath.2021.01535 PMC10518124 34514565

[9] Žulpaitė R , Jagelavičius Ž , Mickys U , Janilionis R . Primary pulmonary meningioma with Rhabdoid features. Int J Surg Pathol. 2019;27(4):457–63. 30563401 10.1177/1066896918819257

[10] Gupta NC , Maloof J , Gunel E . Probability of malignancy in solitary pulmonary nodules using fluorine‐18‐FDG and PET. J Nucl Med. 1996;37(6):943–8. 8683316

[11] Oh JH , Cho HS , Hwang HS , Ji W . Primary pulmonary meningioma presenting as multiple lung nodules: a case report. Thor Cancer. 2022;13:141–3. 10.1111/1759-7714.14270 PMC8720623 34878222

[12] Spinelli M , Claren R , Colombi R , Sironi M . Primary pulmonary meningioma may arise from meningothelial‐like nodules. Adv Clin Pathol. 2000;4(1):35–9. 10936897

